# Identification of Hypoxia-Related Subtypes, Establishment of Prognostic Models, and Characteristics of Tumor Microenvironment Infiltration in Colon Cancer

**DOI:** 10.3389/fgene.2022.919389

**Published:** 2022-06-17

**Authors:** Changjing Wang, Yujie Tang, Hongqing Ma, Sisi Wei, Xuhua Hu, Lianmei Zhao, Guiying Wang

**Affiliations:** ^1^ Department of Gastrointestinal Surgery, The Third Hospital of Hebei Medical University, Shijiazhuang, China; ^2^ The Second Department of Surgery, The Fourth Hospital of Hebei Medical University, Shijiazhuang, China; ^3^ Research Center, The Fourth Hospital of Hebei Medical University, Shijiazhuang, China

**Keywords:** colon cancer, hypoxia-related genes, molecular subtype, tumor microenvironment, immunotherapy, immune checkpoint blockade, HRG-score

## Abstract

**Background:** Immunotherapy is a treatment that can significantly improve the prognosis of patients with colon cancer, but the response to immunotherapy is different in patients with colon cancer because of the heterogeneity of colon carcinoma and the complex nature of the tumor microenvironment (TME). In the precision therapy mode, finding predictive biomarkers that can accurately identify immunotherapy-sensitive types of colon cancer is essential. Hypoxia plays an important role in tumor proliferation, apoptosis, angiogenesis, invasion and metastasis, energy metabolism, and chemotherapy and immunotherapy resistance. Thus, understanding the mechanism of hypoxia-related genes (HRGs) in colon cancer progression and constructing hypoxia-related signatures will help enrich our treatment strategies and improve patient prognosis.

**Methods:** We obtained the gene expression data and corresponding clinical information of 1,025 colon carcinoma patients from The Cancer Genome Atlas (TCGA) and the Gene Expression Omnibus (GEO) databases, respectively. We identified two distinct hypoxia subtypes (subtype A and subtype B) according to unsupervised clustering analysis and assessed the clinical parameters, prognosis, and TME cell-infiltrating characteristics of patients in the two subtypes. We identified 1,132 differentially expressed genes (DEGs) between the two hypoxia subtypes, and all patients were randomly divided into the training group (n = 513) and testing groups (n = 512). Following univariate Cox regression with DEGs, we construct the prognostic model (HRG-score) including six genes (S1PR3, ETV5, CD36, FOXC1, CXCL10, and MMP12) through the LASSO–multivariate cox method in the training group. We comprehensively evaluated the sensitivity and applicability of the HRG-score model from the training group and the testing group, respectively. We explored the correlation between HRG-score and clinical parameters, tumor microenvironment, cancer stem cells (CSCs), and MMR status. In order to evaluate the value of the risk model in clinical application, we further analyzed the sensitivity of chemotherapeutics and immunotherapy between the low-risk group and high-risk group and constructed a nomogram for improving the clinical application of the HRG-score.

**Result:** Subtype A was significantly enriched in metabolism-related pathways, and subtype B was significantly enriched in immune activation and several tumor-associated pathways. The level of immune cell infiltration and immune checkpoint-related genes, stromal score, estimate score, and immune dysfunction and exclusion (TIDE) prediction score was significantly different in subtype A and subtype B. The level of immune checkpoint-related genes and TIDE score was significantly lower in subtype A than that in subtype B, indicating that subtype A might benefit from immune checkpoint inhibitors. Finally, an HRG-score signature for predicting prognosis was constructed through the training group, and the predictive capability was validated through the testing group. The survival analysis and correlation analysis of clinical parameters revealed that the prognosis of patients in the high-risk group was significantly worse than that in the low-risk group. There were also significant differences in immune status, mismatch repair status (MMR), and cancer stem cell index (CSC), between the two risk groups. The correlation analysis of risk scores with IC_50_ and IPS showed that patients in the low-risk group had a higher benefit from chemotherapy and immunotherapy than those in the high-risk group, and the external validation IMvigor210 demonstrated that patients with low risk were more sensitive to immunotherapy.

**Conclusion:** We identified two novel molecular subgroups based on HRGs and constructed an HRG-score model consisting of six genes, which can help us to better understand the mechanisms of hypoxia-related genes in the progression of colon cancer and identify patients susceptible to chemotherapy or immunotherapy, so as to achieve precision therapy for colon cancer.

## Introduction

Colon cancer is the fifth most common malignancy, with more than 1 million new cases every year (Sung et al., 2021). Metastasis and recurrence have always been the main problems leading to refractory colon cancer (Bekaii-Saab et al., 2019; Mayer et al., 2015; Sartore-Bianchi et al., 2016), and about 30–50% of patients with primary colon cancer will relapse and die from metastatic cancer (Arnold et al., 2015; Siegel et al., 2021). Surgical treatment is the main treatment for colon cancer, and the 5-year survival rate is about 50% (Ferlay et al., 2010). The 5-year survival rate for patients with distal metastasis is even worse at about 14%. With the advances in treatments such as surgery, radiation therapy, chemotherapy, and immunotherapy, the survival rate in colon cancer patients has improved significantly (Jahanafrooz et al., 2020). Up to now, the tumor stage has been the most important factor in judging the severity of a patient’s disease, specifying treatment strategy, and predicting the prognosis (Compton et al., 2000).

Moreover, given the high heterogeneity in molecular genetics and histopathology, the treatment strategies based on the tumor-node-metastasis staging system may not be effective across all individuals. With the advance in genomic technology, many epigenetic changes have been identified as potential prognostic biomarkers in colon cancer patients, such as aberrant DNA methylation processes, noncoding RNA and microRNA disorders, and histone modification changes (Kandimalla et al., 2021; Vymetalkova et al., 2019). However, genetic changes still play a key role in the progression of colon cancer. Therefore, the construction of prognostic markers based on changes in genes is vital to enable individualized treatment decisions, which may then guide the choice of treatment strategy and the accurate prediction of patient prognosis.

Tumor cells are metabolically active, so hypoxia often occurs in the center. Hypoxia affects the tumor immune microenvironment (TIME) directly and indirectly, with much evidence favoring an immunosuppressive effect (Chouaib et al., 2018; You et al., 2021). For tumor cells, hypoxia enhances angiogenesis and remodeling by inducing hypoxia-inducible factor (HIF) expression, which is a marker of tumor proliferation, metastasis, and recurrence (King et al., 2021). Potential mechanisms include altered gene expression, oncogene activation, inactivation of anti-oncogenes, decreased genome stability, and clonal selection (Emami Nejad et al., 2021). Under normal oxygen tension, the HIF protein is unstable and easily degraded by proteasome (Semenza et al., 2010; Semenza et al., 2021). In hypoxic cells, HIF proteins are not easily degraded, thereby creating an immune-unfavorable microenvironment by regulating the transcription of downstream genes, ultimately leading to immune resistance (Chouaib et al., 2018; Noman et al., 2019). Hypoxia can regulate the status of the tumor immune microenvironment by promoting the recruitment of innate immune cells and interfering with the differentiation and function of adaptive immune cells (Palazon et al., 2014). For colon cancer, hypoxia also promotes epithelial–mesenchymal transformation (EMT) and ultimately leads to further migration and invasion of tumor cells (Choietal et al., 2017).

In the study, we systematically evaluated the patterns of hypoxia-related genes and tumor immune microenvironment characteristics of COAD patients by clustering the expression of hypoxia genes. We identified two subtypes with distinct clinical and immune characteristics in COAD and constructed an HRG-score signature based on the expression profile of HRGs.HRG-score serves as a reliable predictor of overall survival, clinical characteristics, and immune cell infiltration, which has the potential to be applied as a valuable biomarker for COAD immunotherapy.

## Materials and Methods

### Dataset Collection and Processing

The gene expression data (fragments per kilobase million, FPKM) and the corresponding clinicopathological information of colon carcinoma were downloaded from TCGA-COAD project (https://portal.gdc.cancer.gov/) databases and the GSE39582 cohort (https://www.ncbi.nlm.nih.gov/geo/).

In order to obtain reliable results, samples with no information on survival outcomes were excluded, and a total of 1,025 COAD patients were eventually included in the follow-up analysis. Details of these 1,025 COAD patients are presented in Supplementary Table S1. Before merging the expression matrices of TCGA-COAD project and GSE39582 cohort, the FPKM values of TCGA-COAD were transformed into transcripts per kilobase million (TPM), which were considered to be more comparable with the microarray data. In addition, all raw data were normalized and standardized to eliminate batch effects by using the R software package. Meanwhile, we downloaded the IMvigor210 cohort from the website, which was a cohort study for evaluating the clinical response of atezolizumab in metastatic urothelial cancer (mUC) (Mariathasan et al., 2018). In the IMvigor210 cohort, we excluded the patients with no clinical response information and a total of 298 patients for subsequent validation (Supplementary Table S2). In total, 200 hypoxia-related genes (HRGs) were retrieved from the MSigDB database (http://www.broad.mit.edu/gsea/msigdb/), and the full details of these genes are shown in Supplementary Table S3.

### Consensus Clustering Analysis Based on Hypoxia-Related Genes

Unsupervised clustering analysis was employed to classify patients into distinct molecular subtypes according to the expression of 200 HRGs. In order to increase the intra-class correlation and decrease the correlation, the consensus clustering algorithm was performed and repeated 1,000 times to ensure the stability of the clusters, which we plotted using the R package “ConsensusClusterPlus.”

### Relationship Between Molecular Subtypes With the Clinical Parameters and Prognosis of Colon Carcinoma

We compared the relationships between molecular subtypes, clinical parameters, and prognosis to examine the clinical value of the two subtypes identified by consensus clustering. Furthermore, we also analyzed the expression of the HRGs among the two subtypes. The clinical parameters included age, sex, T stage, N stage, M stage, and TNM stage. Kaplan–Meier curves were used to assess the differences in overall survival among different molecular subtypes.

### Molecular and Immune Features Between Subtypes

GSVA enrichment analysis was employed to assess and compare the difference in biological pathways between the distinct molecular subtypes. and the hallmark gene set (c2. cp.kegg.v7.2) was retrieved from the MSigDB database. Meanwhile, we estimated the relative abundance of 23 immune cells in colon carcinoma using a single-sample gene set enrichment analysis (ssGSEA) algorithm, which was performed using the GSVA R package.

Considering the role of the tumor microenvironment (TME) in tumor progression, we also evaluated the Stromal, Immune, and ESTIMATE scores of each sample by the ESTIMATE algorithm to determine the degree of immune cell infiltration of each subtype. We not only estimated and compared the expression level of six common immune checkpoint-related genes, such as CD274 (PD-L1), PDCD1LG2 (PD-L2), PDCD1 (PD-1), CTLA4, LAG3, and TIGI, but also calculated the patient TIDE score to evaluate the immunotherapy response.

### Identification of Differentially Expressed Genes

DEGs between the two hypoxia-related subtypes were identified using the “limma” R package, and the significance criterion for defining DEGs was |log fold change (FC)| > 0.585 and adjusted *p*-value < 0.05. Furthermore, we performed Gene Ontology (GO) and Kyoto Encyclopedia of Genes and Genomes (KEGG) enrichment analysis on DEGs to identify the related gene functions and enriched pathways through the “clusterProfiler” R package with a cut-off *p* value < 0.05 and an adjusted *p* value < 0.05.

### Construction of the Prognostic Hypoxia-Related Gene Score

First, univariate Cox regression analysis was performed on DEGs to identify those linked to the prognostic value with a *p*-value < 0.05. Second, a total of 1,025 patients were randomly categorized into the training group (n = 513) and testing group (n = 512) at a ratio of 1:1; then, the patients in the training group were used to construct the hypoxia-related prognostic HRG-score, and the testing group was used for validation. Finally, based on hypoxia-related prognostic DEGs, The LASSO–Cox regression analysis was then utilized to develop the prognostic HRG-score in the training group, which was performed using the “glmnet” R packet. The HRG-score formula is as follows: HRG-score = Σ (Expi * Coefi), where Coefi and Expi denote the risk coefficient and expression of each gene, respectively. Based on the HRG-score formula, each patient can get a specific risk score. A total of 513 patients in the training group were assigned, based on a median value, to the high-risk group (n = 256) and low-risk group (n = 257). Similarly, a total of 512 patients in the testing group were assigned to HRG-score-related subgroups based on the formula constructed by the training group. The receiver operating characteristic (ROC) curve, which is used to judge the accuracy of the prognostic risk model, was generated by the “timeROC” R package, and principal component analysis (PCA) was performed using the “ggplot2” R package.

RNAseq data (level3) and the corresponding clinical information for 450 colon cancer tumors were obtained from The Cancer Genome Atlas (TCGA) dataset (https://portal.gdc.com). First univariate and multivariate cox regression analyses and forest plots were used to display each variable (*p*-value, HR, and 95% CI) via the “forest plot” package. Based on the results of multivariate Cox proportional risk analysis, column line plots were created using the “rms” package to predict the total recurrence rate in 1, 2, and 3 years. The line graphs provide graphical results for these factors, allowing the prognostic risk of individual patients to be calculated by the points associated with each risk factor.

### Correlation Analysis of the HPR-Score With Clinical Parameters

A Chi-square test was applied to explore the correlation between the HRG-score and the clinical parameters (age, gender, T stage, N stage, M stage, and TNM stage). To assess whether the HRG-score is an independent prognostic factor associated with prognosis, we performed univariate analysis and multivariate analysis on the training group and testing group. Kaplan-Meier analysis was used to compare survival outcomes of patients between high- and low-risk and assessed the correlation between the survival outcome and HRG-score. We further analyzed the relationship between HRG-score and molecular subtypes through a boxplot.

### Evaluation of Immune Status and Mismatch Repair Status Between the High- and Low-Risk Groups

The CIBERSORT algorithm was used to calculate the relative abundance of 22 infiltrating immune cells per sample in the low- and high-risk groups (Supplementary Table S11). We explored the correlation between the 22 infiltrating immune cell fractions and the 7 genes in the PRG scores. In addition, we compared the expression levels of immune checkpoints between the low- and high-scoring groups and analyzed the relationship between the HRG score and the cancer stem cell (CSC) index.

### Sensitivity Analysis of Chemotherapy and Immunotherapy

In a project to evaluate the difference in the treatment effect of five chemotherapeutic agents in patients in the high-risk and low-risk groups, the semi-inhibitory concentration (IC_50_) values of chemotherapeutic agents were analyzed by the R package “pRRophetic.” We acquired the IPS of colon cancer patients in TCGA-COAD project from TCIA database and compared the IPS of the distinct risk group to evaluate the response to immune checkpoint-blocking therapy. We further explored the relationship between immunotherapy sensitivity and HRG-score by the IMvigo210 cohort.

### Statistical Analysis

All statistical analyses were performed using R software (v4.0.2). *p*-values <0.05 were considered statistically significant if not explicitly stated.

## Result

### Identification of Hypoxia Gene-Related Subtypes in Colon Carcinoma

**FIGURE 1:**
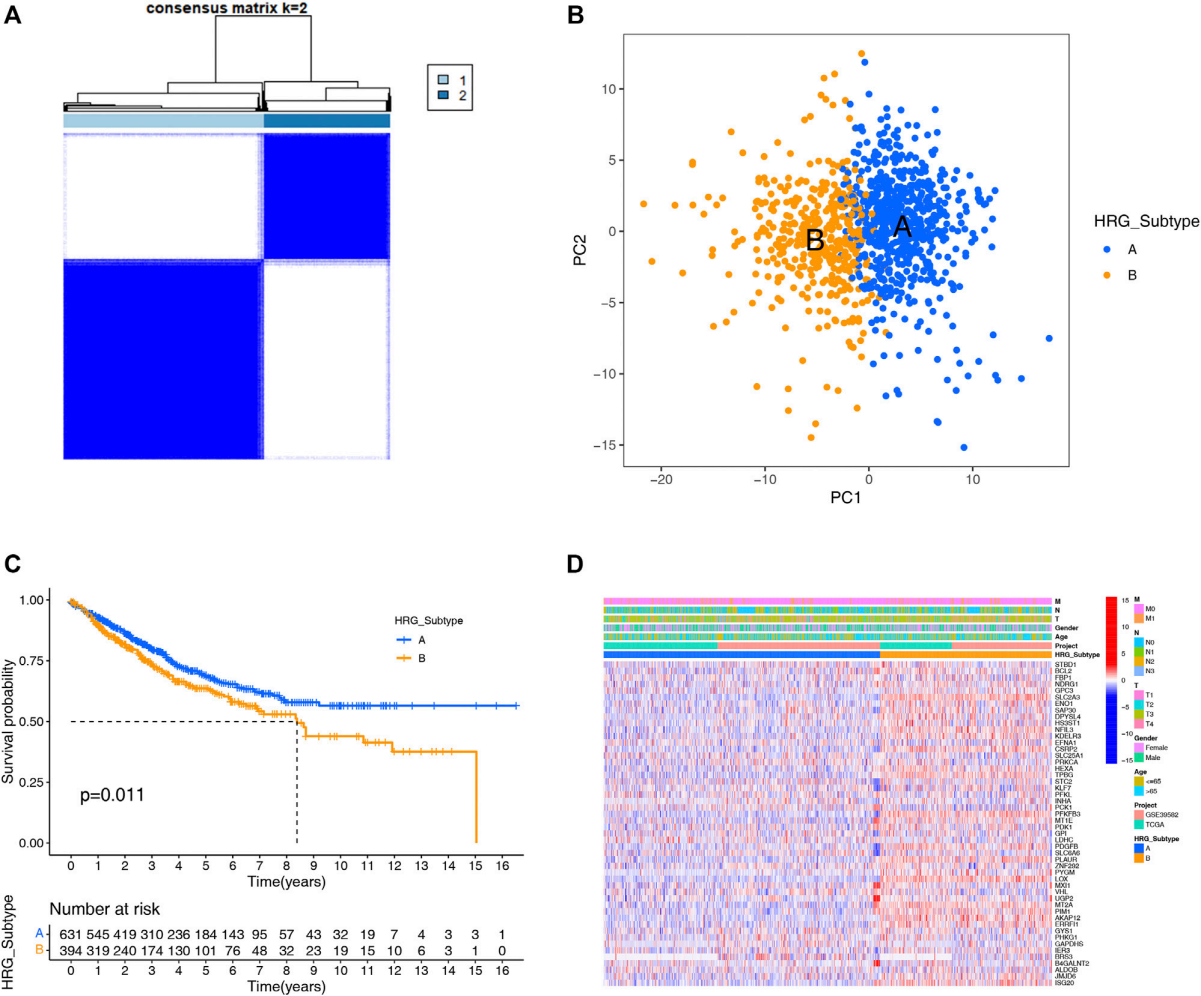
HRG subtypes and clinical parameters and biological characteristics of two distinct subtypes of samples divided by consistent clustering. **(A)** Consensus matrix heatmap defining two subtypes (k = 2) and their correlation area. **(B)** PCA showing a remarkable difference in transcriptomes between the distinct HRG-subtypes, and each dot represents a single sample. **(C)** KM survival curve analysis showed that the overall survival time of the distinct HRG-subtypes was different (log-rank tests, *p* < 0.001). **(D)** Differences in clinical parameters and HRG expression levels between the two distinct HRG-subtypes.

A total of 1,025 patient samples with complete survival information from TCGA-COAD project and GEO-GES39582 were included in our study. To further investigate the expression characteristics of HRGs in colon carcinoma, we used a consensus clustering algorithm to cluster the patients based on the expression of the 200 HRGs. Our result found that when K = 2, the intra-group correlations were the highest, and the inter-group correlations were the lowest, indicating sorting the entire patients into two subtypes may be the most optimal selection (Figure 1A). PCA analysis revealed the significant differences between the two subtypes (Figure 1B), suggesting there existed significant heterogeneity in the expression of hypoxia genes in patients with colon carcinoma. The Kaplan–Meier curves showed an obvious difference in the prognosis between the two hypoxia subtypes, and the prognosis in patients with subtype A was significantly better than that in patients with subtype B (log-rank test, *p* = 0.011; Figure 1C). Furthermore, we compared the correlations of the two subtypes with clinical parameters and the expression of hypoxia genes. As the heatmap showed (Figure 1D), there were no significant differences in clinical parameters between the two subtypes; however, compared with subtype A, most of the hypoxia-related genes were highly expressed in subtype B.

### Function Enrichment of the Molecular Subtypes

**FIGURE 2:**
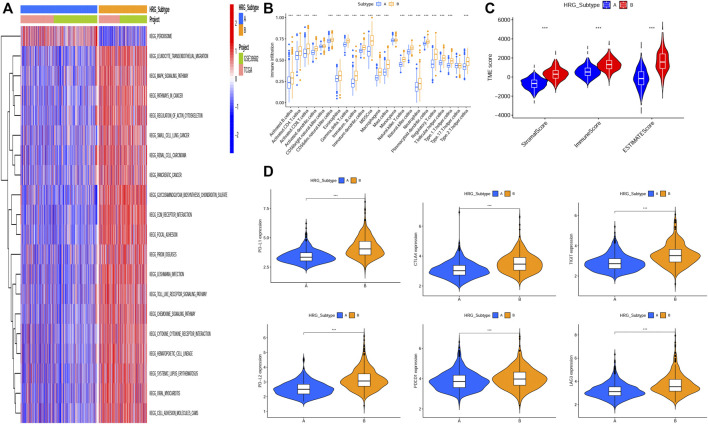
Correlations of tumor immune cell microenvironments and two HRG-subtypes. **(A)** GSVA of biological pathways between two distinct subtypes, in which red and blue represent activated pathways and blue represents inhibited pathways, respectively. **(B) **Relative abundance of 23 infiltrating immune cell types in the two HRG-subtypes. **(C)**Correlations between the two CRC subtypes and TME score. **(D–I)** Expression levels of PD-L1, PD-L2, PDCD1, LAG3, TIGIT, and CTLA4 in two distinct HRG-subtypes. (*p < 0.05; **p < 0.01; ***p < 0.001).

GSVA enrichment analysis showed that metabolism-related and DNA synthesis-related pathways including butanoate metabolism, propanoate metabolism, pyruvate metabolism, fatty acid metabolism, nonhomologous end joining, base excision repair, DNA replication-related pathway were upregulated in subtype A, while T- and B-cell receptor signaling pathway, natural killer cell-mediated cytotoxicity, antigen processing and presentation, checkpoint signaling pathway, and NOD-like, RIG-I-like, and Toll-like receptor signaling pathways were upregulated in subtype B(Figure 2A, Supplementary Table S4).

### Characteristics of the Tumor Microenvironment in Distinct Subtypes

The tumor microenvironment (TME) has been proved to play an important role in tumor progression and immune response. We evaluated the 23 immune cells’ infiltration levels of each patient by applying the ssGSEA (Supplementary Table S5) and found significant differences in the infiltration of most immune cells between the two subtypes (Figure 2B). The infiltrate levels of 20 immune cell types, including activated B cells, activated CD4^+^ T cells, activated CD8^+^ T cells, natural killer T cells, and regulatory T cells, were significantly higher in the subtype B than those in the subtype A. The ESTIMATE algorithm was used to evaluate the TME score (stromal score, immune score, and ESTIMATE score) of each patient through the “ESTIMATE” R package (Supplementary Table S6), and we found that the stromal score, immune score, and ESTIMATE score were significantly higher in subtype B than subtype A (Figure 2C). Recently, the immune checkpoint blockade has achieved promising results in the immunotherapy of tumors. Therefore, we subsequently analyzed the expression levels of several important immune checkpoint-related genes, such as CD274 (PD-L1), PDCD1LG2, PDCD1, CTLA4, LAG3, and TIGIT (Figures 2D–I). We found that the expression levels of six immune checkpoint-related genes in subtype B were higher than those in subtype A, indicating that patients in subtype B were more likely to form an immunosuppressive microenvironment and escape from immune surveillance.

### Construction and Validation of the Prognostic Hypoxia Related Gene-Score

**FIGURE 3:**
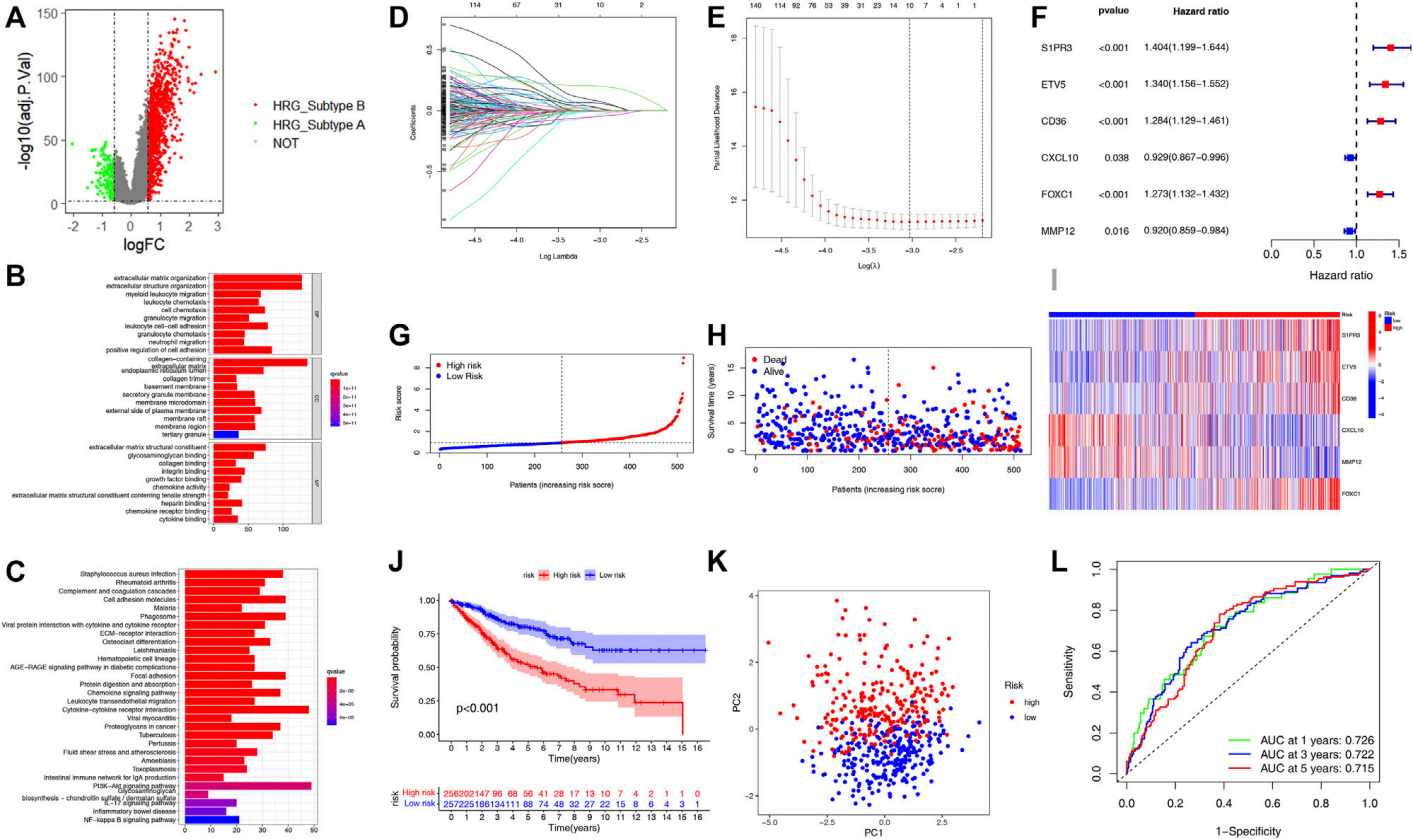
Construction of the HRG-score in the training set based on the differentially expressed genes of two distinct HRG-subtypes. **(A)** Volcano plot of differentially expressed genes between the two distinct HRG-subtype. Gray dots represent not significant genes, green dots represent upregulated genes in HRG-subtype A, and red dots represent upregulated genes in HRG-subtype B **(B–C)** GO and KEGG enrichment analyses of DEGs among two distinct HRG-subtypes. **(D–E)** LASSO regression analysis and partial likelihood deviance on the prognostic genes. **(F)** Forest plot of multivariate cox regression analysis for prognostic genes. **(G–H)** Ranked dot and scatter plots showing the HRG-score distribution and patient survival status. **(I)** Heatmap of the expression of six genes involved in the HRG-score in low- and high-risk groups. **(J)** Survival analysis of the patients in low- and high-risk groups. **(K)** PCA based on the prognostic signature. (L) ROC curves to predict the sensitivity and specificity of 1-, 3-, and 5-year survival according to the HRG-score.

We identified 1,132 DEGs between the two HRG-related subtypes, of which 139 genes were upregulated in subtype A and 993 genes were upregulated in subtype B (Figure 3A, Supplementary Table S7). Then, we conducted GO and KEGG enrichment analysis on the 1,132 DEGs to explore the potential function and pathway through the “clusterProfiler” R package. In the GO analysis, the top 5 most significantly enriched terms were collagen-containing extracellular matrix, extracellular matrix organization, extracellular structure organization, positive regulation of cell adhesion, and negative regulation of immune system process (Figure 3B, Supplementary Table S8).In the KEGG analysis, the top 5 most significantly enriched terms were PI3K-Akt signaling pathway, cytokine–cytokine receptor interaction, cell adhesion molecules, phagosome, and focal adhesion (Figure 3C, Supplementary Table S9). Univariate Cox regression analysis was employed on the 1,132 DEGs and 437 genes associated with the prognostic value with a *p*-value <0.05 and were identified as candidate genes for subsequent analysis (Supplementary Table S10). Then, all patients were classified into training group (n = 513) and testing group (n = 512) at a ratio of 1:1 randomly, the training group for developing the prognostic signature and the testing group for validation. LASSO regression analysis on the 437 candidate genes was performed to exclude overlapping genes and reduce the fitting effect of the signature (Figures 3D,E). Finally, six genes were included to construct the risk model after multivariate Cox proportional risk regression analysis, four of which were associated with high risk and two with low risk (Figure 3F). According to the results of the multivariate Cox proportional risk regression analysis, the HRG-score was constructed as follows: Risk score = (0.2665 * expression of S1PR3) + (0.2478* expression of ETV5) + (0.2115* expression of CD36) + (0.2808* expression of FOXC1) + (−0.1735* expression of CXCL10) + (−0.0976* expression of MMP12). According to the median risk score, patients in the training group were classified into high-risk group (n = 256) and low-risk group (n = 257) (Figure 3G). When compared to the low-risk group, we found that more patients died and a shorter survival time in the high-risk group (Figure 3H). The expression levels of six genes involved in the construction of our HRG-score signature are shown in Figure 3I. Kaplan–Meier survival analysis revealed that there existed a significant difference in survival time between the low- and high-risk group, and the patients in the low-risk group had a longer survival time (*p* < 0.001) (Figure 3J). The principal component analysis (PCA) showed that patients with different risks were well separated into two clusters (Figure 3K). The AUC values for the 1-, 3-, and 5-year survival were 0.726, 0.722, and 0.715, respectively (Figure 3L).

### Validation of the Hypoxia Related Gene-Score Signature

**FIGURE 4:**
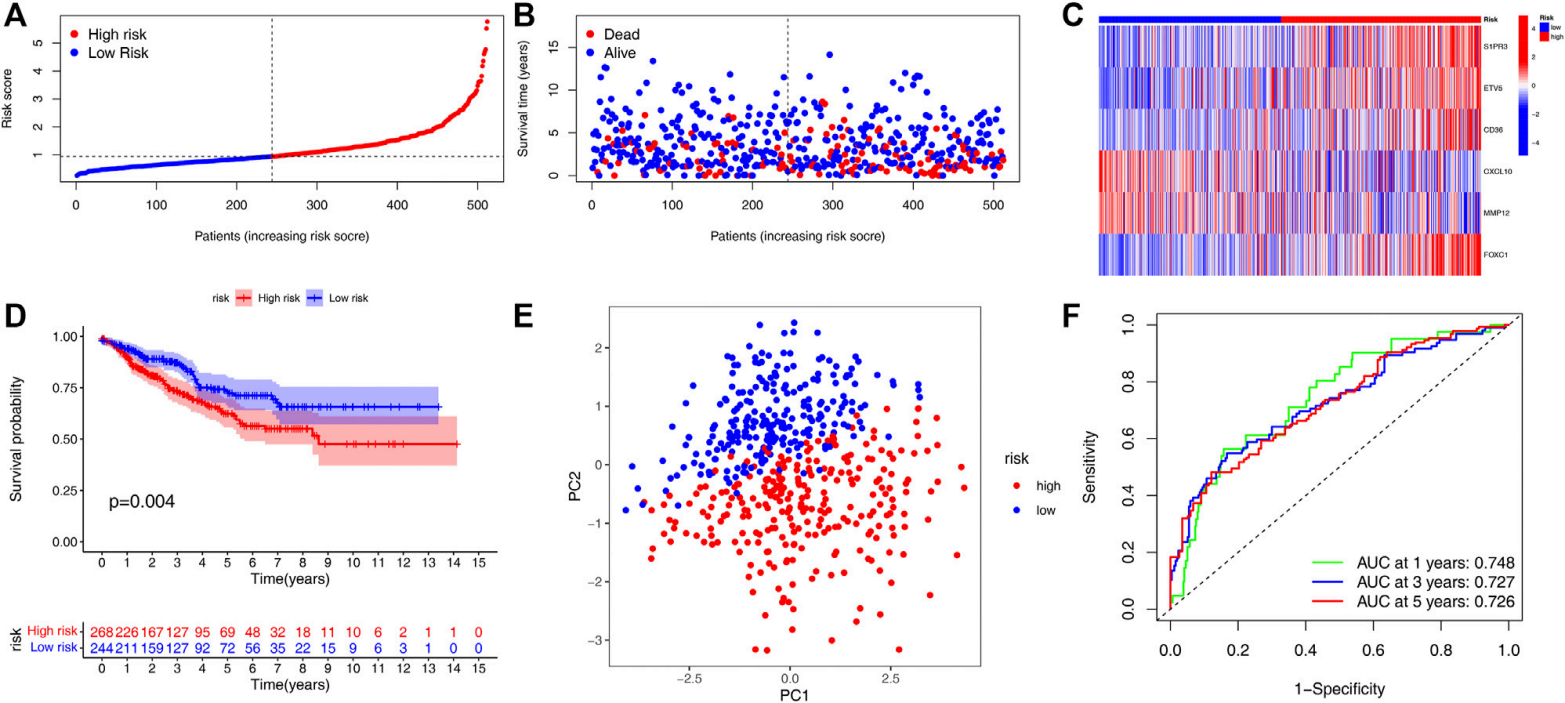
Validation of the HRG-score signature in the testing set. **(A,B)** Ranked dot plot indicates the PRG-score distribution, and the scatter plot presents the patients’ survival status. **(C)** Heatmap of the expression of six genes involved in the HRG-score in low- and high-risk groups. **(D)** KM analysis of the OS between the low- and high-risk groups. **(E)** PCA demonstrated that the patients in the different risk groups were distributed in two directions. **(F)** ROC curves to predict the sensitivity and specificity of 1-, 3-, and 5-year survival according to the PRG-score.

In order to verify the practicality and credibility of the model, we performed the same analysis for internal validation using a testing group (n = 512). Based on the median risk score in the training group, all patients in the testing group were classified into the low-risk group (n = 244) and high-risk group (n = 268) (Figure 4A). Compared to the low-risk group, the proportion of patient deaths tended to be high in the high-risk group (Figure 4B). Heatmap was also plotted to analyze the expression of the six genes involved in the HRG-score signature between the high- and low-risk groups (Figure 4C). Kaplan–Meier analysis showed that the survival probability of the high-risk group was significantly lower than that of the low-risk group (*p* < 0.04) (Figure 4D). The principal component analysis (PCA) showed that the patients with different risk scores can be stratified into two clusters distinctly (Figure 4E). The AUC values for the 1, 3, and 5 years of ROC were 0.748, 0.727, and 0.726 respectively, indicating our model's good predictive efficacy (Figure 4F). Nomograms of S1PR3, ETV5, CD36, FOXC1, CXCL10, and MMP12 expression and independent clinical risk factors (age and pathological stage) were constructed (Supplementary Figure S4). A higher total number of points in the nomogram represents a worse prognosis. In addition, the C-index value was 0.779 (*p* < 0.001). The deviation-corrected line in the calibration plot was close to the ideal curve (i.e., 45° line), indicating good agreement between the predicted and observed results.

### Correlation Analysis of Hypoxia Related Gene-Score and Clinical Parameters

**FIGURE 5:**
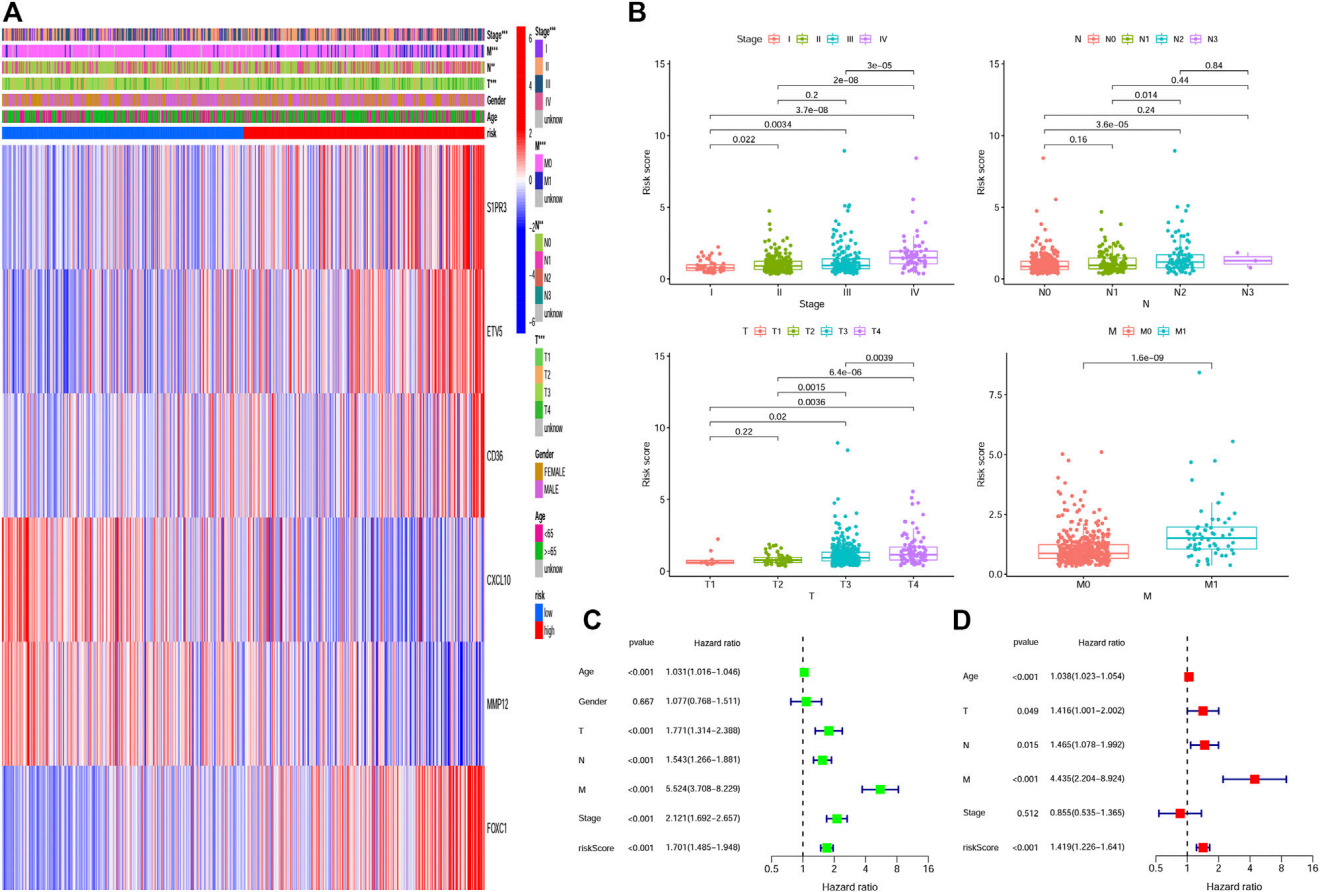
Correlation and independent prognosis analysis of HRG-score and clinical parameters in the training set. **(A,B)** Univariate and multivariate analyses of the prognostic value of the HRG-score. **(C)** Relationships between clinical parameters and the low- and high-risk groups. **(D)** Clinical application value of HRG-score in predicting T stage, N stage, M stage, and TNM stage, respectively (*p < 0.05; **p < 0.01; ***p < 0.001).

We plotted a heatmap of clinical parameters for the patients in the training group and found statistically significant differences in T, N, M, and TNM stages between high- and low-risk groups (Figure 5A). We further analyzed the relationship between the T stage, N stage, M stage, and TNM stage and risk score separately. As shown in Figure 5B, we found significant differences in risk scores for T, N, M, and TNM stages, and patients’ clinical stage deteriorated as risk scores increased, suggesting that high-risk scores predicted poor outcomes for patients. In addition, we also analyzed the correlation between the risk score of the testing group and the clinical parameters (Supplementary Figure S1A) and obtained the same result that the risk score can be used to evaluate the prognosis of patients (Supplementary Figure S1B–E). Univariate and multivariate Cox regression analyses were employed to assess whether HRG-score could be used as an independent prognostic factor. The univariate Cox regression analysis indicated that the HRG-score was an independent factor predicting poor survival in the training group (HR = 1.701, 95% CI: 1.485–1.948) (Figure 5C). After adjusting for other confounding factors, the multivariate analysis yielded similar results that the HRG-score can be a prognostic factor for patients in the training group (HR = 1.419, 95% CI: 1,226–1.641) (Figure 5D). Univariate and multivariate Cox regression analyses were also employed in the testing group, and we also got the same result (HR = 1.505, 95% CI: 1.344–1.686 and HR = 1.297, 95% CI: 1.147–1.467, Supplementary Figure S2A,B).

### Evaluation of Tumor Microenvironment and Checkpoints Between the High- and Low-Risk Groups

**FIGURE 6:**
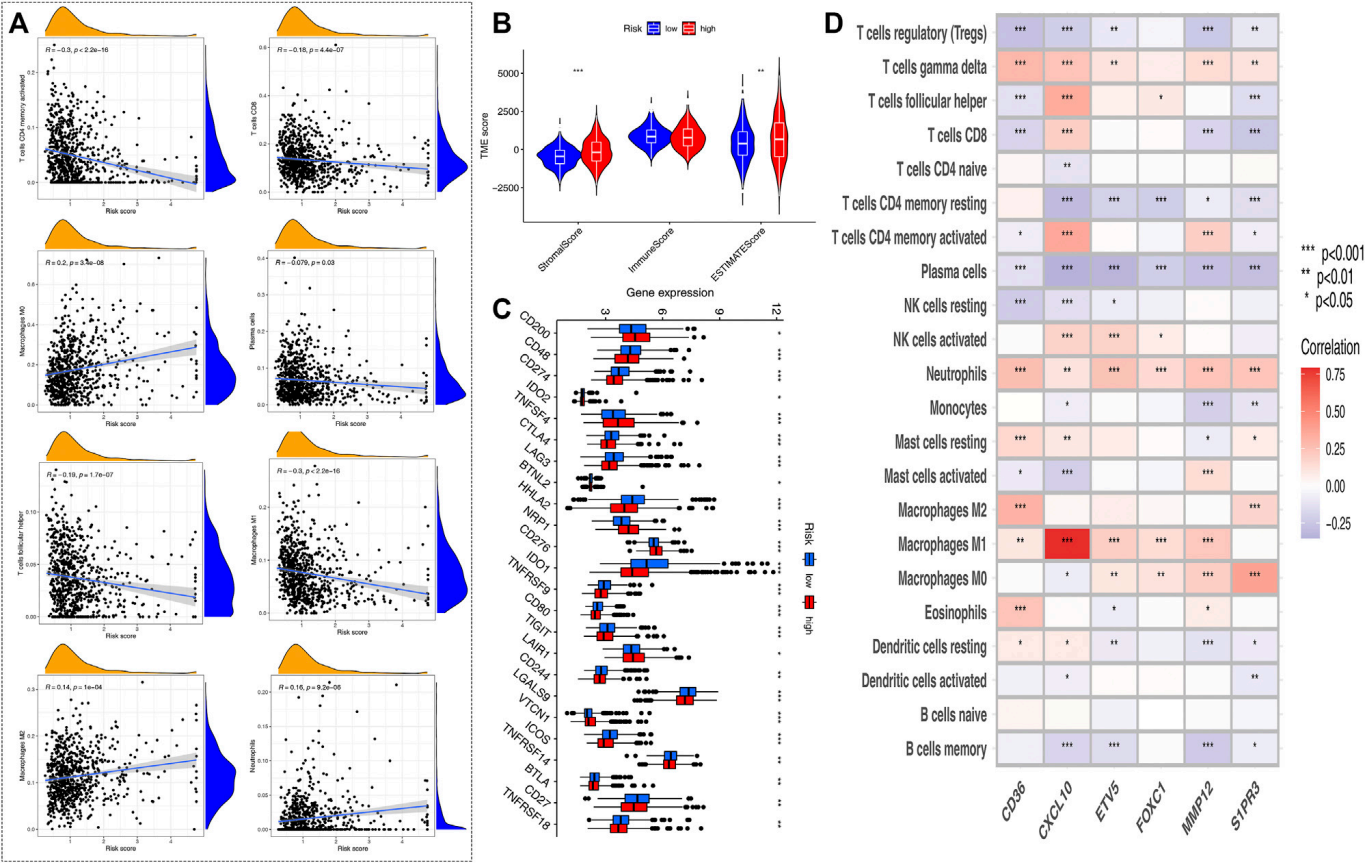
Evaluation of the TME and checkpoints between the two risk groups. **(A)** Correlations between HRG-score and immune cell types. **(B)** Correlations between HRG-score and TME score. **(C)** Expression of immune checkpoint-related genes in the low- and high-risk groups. **(D)** Correlations between the relative abundance of immune cells and six genes involved in the HRG-score. (*p < 0.05; **p < 0.01; ***p < 0.001)

CIBERSORT algorithm was performed to assess the association between the HRG-score and the abundance of immune cells. The scatter diagrams showed that the HRG-score was positively correlated with macrophage M2, neutrophils, and macrophages M0 and negatively correlated with macrophages M1, plasma cells, T cell CD4 memory activated, T-cell follicular helper, and T cell CD8 (Figure 6A). We observed that the stromal score and ESTIMATE score were significantly higher in the low-risk group than the high-risk group (Figure 6B). Figure 6C shows that 22 immune checkpoints were differentially expressed in the two groups, and the expression of most immune checkpoint-related genes was higher in the low-risk group than that in the high-risk group. We also assessed the correlation between the six genes of the HRG-score signature and the abundance of immune cells. We observed that most immune cells were significantly correlated with the six genes (Figure 6D).

### Correlation Analysis of PRG-Score With the MMR Status and CSC Index

**FIGURE 7:**
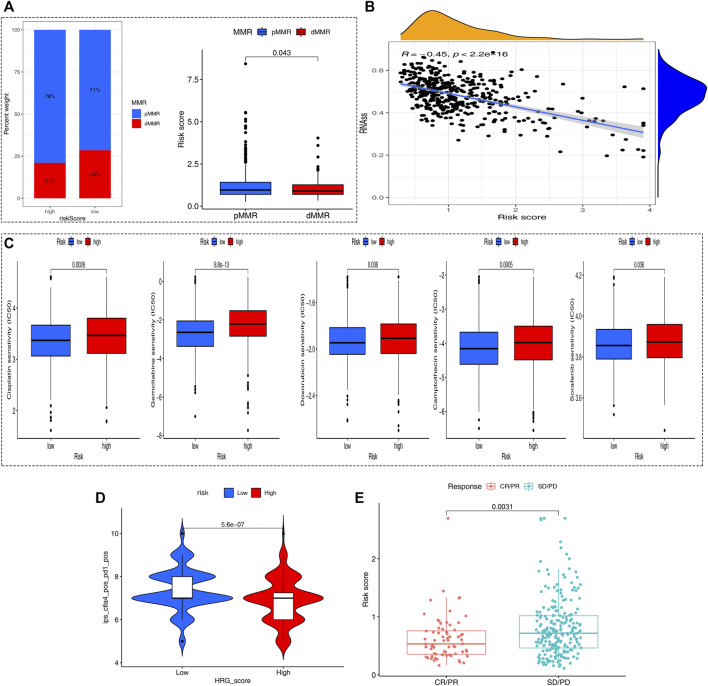
Comprehensive analysis of the HRG-score in COAD. **(A)** Relationships between the HRG-score and MMR status. **(B)** Relationships between the HRG-score and CSC index. **(C)** Relationships between HRG-score and sensitivity of five chemotherapeutics. **(D)** Prediction of the response of different risk samples to the combination of anti-CTLA4 and anti-PD1 based on IPS. **(E)** Boxplot for assessing HRG-score in predicting anti-PD-L1 response through the IMvigor210 cohort.

Inactivating mutations in mismatch repair genes such as MLH1, MSH2, MSH6, and PMS2 can cause mismatch repair (MMR) dysfunction and then lead to microsatellite high instability (MSI-H). Patients with high microsatellite instability (MSI-H) are more sensitive to immunotherapy and can benefit from immunotherapy drugs. Correlation analyses revealed that a high HRG-score was significantly correlated with proficient mismatch repair status (pMMR), while a low HRG-score was associated with deficient mismatch repair (dMMR) status (Figure 7A), suggesting that patients with low-risk scores benefit from immunotherapy better than those with high-risk scores. Stem cells (CSCs) are a small subset of undifferentiated cells in tumor tissues, which have strong self-renewal potential and tumorigenic potential, and can form tumors in a low number *in vivo*. The correlation analysis between the PRG-score and CSC index showed that PRG-score was negatively correlated with the CSC index (R = −0.31, *p* < 0.001), indicating that tumor cells with lower HRG-score had a lower degree of cell differentiation and distinct stem cell properties (Figure 7B).

### Analysis of the Sensitivity of Chemotherapeutics and Immunotherapy Based on Hypoxia Related Gene-Score

We next selected four chemotherapy drugs currently used for the treatment of colon carcinoma to assess the sensitivity of patients in the low- and high-risk groups to these drugs. As shown in Figure 7C, we found that the patients in the low-risk group showed more sensitivity to chemotherapy drugs indicating that the low-risk group may benefit more from chemotherapy drugs. Meanwhile, the applicability of different HRG-score samples to combined therapy of anit-CTLA4 and anti-PD1 was compared by IPS. The analysis showed a significant difference (*p* = 0.00023 < 0.05) that the IPS of the low-risk group treated with the combination of anti-CTLA4 and anti-PD1 was relatively higher than that of the high-risk group, indicating that the patients with low HRG-score had a better therapeutic effect on Immunotherapy (Figure 7D). To further evaluate the robustness of our HRG-score signature, we calculated the risk score of patients in the IMvigor210 cohort based on the formula of HRG-score and analyzed the correlation of risk score with the effect of immunotherapy. As shown in Figure 7E, there existed significant differences in risk scores between the complete remission/partial remission (CR/PR) group and stable disease/progressive disease (SD/PD) group, and the risk score of patients in the CR/PR group was significantly lower than that of patients in the SD/PD group (*p* = 0.0031 < 0.05). To further improve the clinical application of our model, we constructed a nomogram containing HRG-score and clinical parameters to predict overall survival at 1, 3, and 5 years (Supplementary Figure S3A), and the calibration plots suggested that the nomogram had a good performance in predicting the survival of colon cancer patients (Supplementary Figure S3B).

## Discussion

CRC is an extremely common malignant tumor. In recent years, there is a tendency to develop to the right half of the colon, which is closely related to heredity, living habits, and colorectal adenoma (Zhang et al., 2020). According to the latest data, the global incidence rate of CRC is the second only to breast cancer and lung cancer, and the mortality rate is the second only to lung cancer. At present, the main treatment of CRC is surgical treatment, supplemented by neoadjuvant radiotherapy and chemotherapy, postoperative radiotherapy and chemotherapy, and immunotherapy. The main prognostic key issues affecting CRC are currently the need for timely surgical intervention and effective radiotherapy treatment. Unfortunately, more than 50% of CRC patients experience tumor recurrence, metastasis, invasion, and resistance to chemotherapy drugs at the time of diagnosis or during their follow-up treatment (Song et al., 2021), thus losing the standard of care of surgical treatment with radiotherapy and subsequently having a poor prognosis as well as poor quality of survival. Chemotherapy is a relative option for patients with CRC who cannot tolerate surgical intervention; however, there are still no specific chemotherapeutic agents for CRC. A growing body of evidence suggests that multiple genes and cellular pathways are involved in the development of CRC. To date, the lack of knowledge about the exact molecular mechanisms underlying CRC progression has limited the ability to treat advanced diseases. Therefore, it is necessary to identify the key genes and pathways of CRC in order to understand its molecular mechanism, explore potential biomarkers, and develop more effective diagnostic and therapeutic strategies.

Hypoxia-inducible factor (HIF) played an important role in cancer biology, including angiogenesis, cell survival, glucose metabolism, and invasion (Zhang et al., 2021). HIF can facilitate metabolic metastasis and enhance the non-mitochondrial mechanism of ATP production, thus providing energy for tumor cells (Gatenby et al., 2004). In addition, HIF stabilization can lead to inhibition of apoptotic pathways through silencing of mitochondrial activity. Hypoxia can mitigate the infiltration rate of immune cells and their function in the TME (You et al., 2021). Glycolysis can lead to acid TME with a pH as low as 5.8 to 6.5, and the acidic environments can inhibit immune cell differentiation and function. With the advance of high-throughput sequencing, identification of molecular characterization gradually becomes a significant method for biomedical research, which can be used for identifying biomarkers for prognosis predicting, recurrence monitoring, and clinical risk stratification (Wang et al., 2009; Xiao et al., 2018).

The growth and progression of malignant tumors are associated with immunosuppression, and tumor cells evade immune surveillance through different mechanisms, including the activation of immune checkpoints pathways that suppress anti-tumor immune responses. The successful development of immune checkpoint genes (ICGs) was a milestone event in tumor immunotherapy and was named one of the top 10 scientific discoveries by Nature in 2013 (Wolchok et al., 2014). ICGs inhibit and kill tumor cells by enhancing the body's anti-tumor immune function and have shown significant clinical efficacy in the treatment of a variety of malignancies, becoming an important tool in tumor therapeutics (Wang et al., 2018). Based on the expression of 120 hypoxia-related genes, 1,025 colon cancer samples from TCGA-COAD project and GEO-GSE39582 were separated into two heterogeneous subtypes, with significant differences in OS between the two subtypes. Hypoxia is an important factor in the poor prognosis of tumor by regulating cancer hallmark, thus creating physical barriers conducive to tumor survival (Abou Khouzam et al., 2022). We found most hypoxia-related genes are highly expressed in subtype B, and the patients in subtype B had a worse survival outcome than those in subtype A. We then compared the several expression levels of six known immune checkpoint genes (PD-L1, PD-L2, PD-1, LAG3, TIGIT, IDO1, and CTLA-4)between the two subtypes, and the expression level of the six genes was significantly higher in subtypes B than subtype A. The previous studies reported that the high expression level of immune checkpoint genes was more likely to form an immunosuppressive microenvironment and promote tumor immune escape (Dunn et al., 2022); meanwhile, the upregulation of immune checkpoint genes (ICGs) was positively correlated with high immune cell infiltration (Hu et al., 2021). The TME score and immune cell infiltration have been reported to be tightly associated with the immunotherapy of cancers and the prognosis (Luo et al., 2020). Thus, we also analyzed the relationship between subtype and immune cell infiltration. Compared with subtype A, the expression level of most immune cells including activated B cells, activated CD4^+^T cells and activated CD^+^8 T cells was significantly higher in subtype B. In addition, we also observed that the stromal score and ESTIMATE score were higher in B than A. These results suggest that patients in subtype A may benefit from immune checkpoint inhibitor therapies. TIDE comparison between the two groups showed that patients with subtype B were more likely to form immune escape than patients with subtype A, which further confirmed our previous results.

Our findings suggest that hypoxic genes differ in the course of changes in the colon. Therefore, we constructed a robust and effective prognostic HRG-score and validated its predictive ability. We explored the expression level of six genes of our HRG-score and found a significant difference between the risk groups. There were significant differences in clinical parameters, prognosis, TME, ICGs, MMR status, CSC index, and drug sensitivity between low- and high-risk HRG-score patients. It will help to better understand the molecular mechanism of colorectal cancer and provide new ideas for targeted therapy (Bai et al., 2020; Huo et al., 2021; Yan et al., 2021).

Immunotherapy is a promising method in cancer treatment and has achieved remarkable efficacy in the treatment of colorectal cancer (Ganesh et al., 2019). Due to the high heterogeneity of molecular genetics and histopathology of colon cancer, immunotherapy still has limitations and obstacles (Makaremi et al., 2021). TME plays a crucial role in the tumorigenesis and progression of COAD, and the immunosuppressive function is one of the causes of poor response to treatment. Immune cells of TME are involved in tumor suppression and progression. Immune-infiltrating cells in TME are mainly composed of dendritic cells, macrophages, NK cells, T cells, and B cells (Koi et al., 2017). Surveillance and elimination of abnormal antigens is an essential feature of the normal function of the immune system. Macrophages and NK cells play a crucial role in stimulating the adaptive immune system that targets tumor cells (Markman et al., 2015), and a higher level of NK cells and CD8^+^ T-cell infiltration often predicts a better prognosis (Sconocchia et al., 2014). In our study, we discovered that the relative abundance of B cells, CD8^+^T cells, NK cells, and macrophages cells was significantly higher in the low-risk group.

In summary, this study conducted a comprehensive bioinformatic analysis of two new molecular subgroups of hypoxic genes and colorectal cancer patients and constructed an HRG-score model consisting of six genes. However, due to the limitations of bioinformatics analysis, further clinical sample testing and cellular and animal experiments are needed to explore the function of hypoxia genes in colorectal cancer and the related molecular mechanisms in depth.

## Data Availability

The datasets presented in this study can be found in online repositories. The names of the repository/repositories and accession number(s) can be found in the article/Supplementary Material.

## Supplementary Material

Supplementary Figure S1 Nomograms and calibration plots predicting the probability of 1-year, 2-year, and 3-years DFS in COAD patients.

Supplementary Table S1 Clinical and prognostic information of 1,025 patients.

Supplementary Table S2 298 patients inside IMvigor120 for follow-up analysis.

Supplementary Table S3 200 hypoxia genes.

Supplementary Table S4 GSVA analysis results.

Supplementary Table S5 ssGSEA calculates the content of 23 immune cells.

Supplementary Table S6 TME SCORE score.

Supplementary Table S7 1,132 differential genes.

Supplementary Table S8 GO analysis.

Supplementary Table S9 KEGG analysis.

Supplementary Table S10 437 one-way prognostic genes.

Supplementary Table S11 CIBERSORT algorithm calculates the relative infiltration content of 22 immune cells.
