# Supplementary figures and images for: Identification of Hypoxia-Related Subtypes, Establishment of Prognostic Models, and Characteristics of Tumor Microenvironment Infiltration in Colon Cancer
Source: Front Genet. 2022 Jun 17;13:919389. doi: 10.3389/fgene.2022.919389 (PMC9247151; doi:10.3389/fgene.2022.919389)

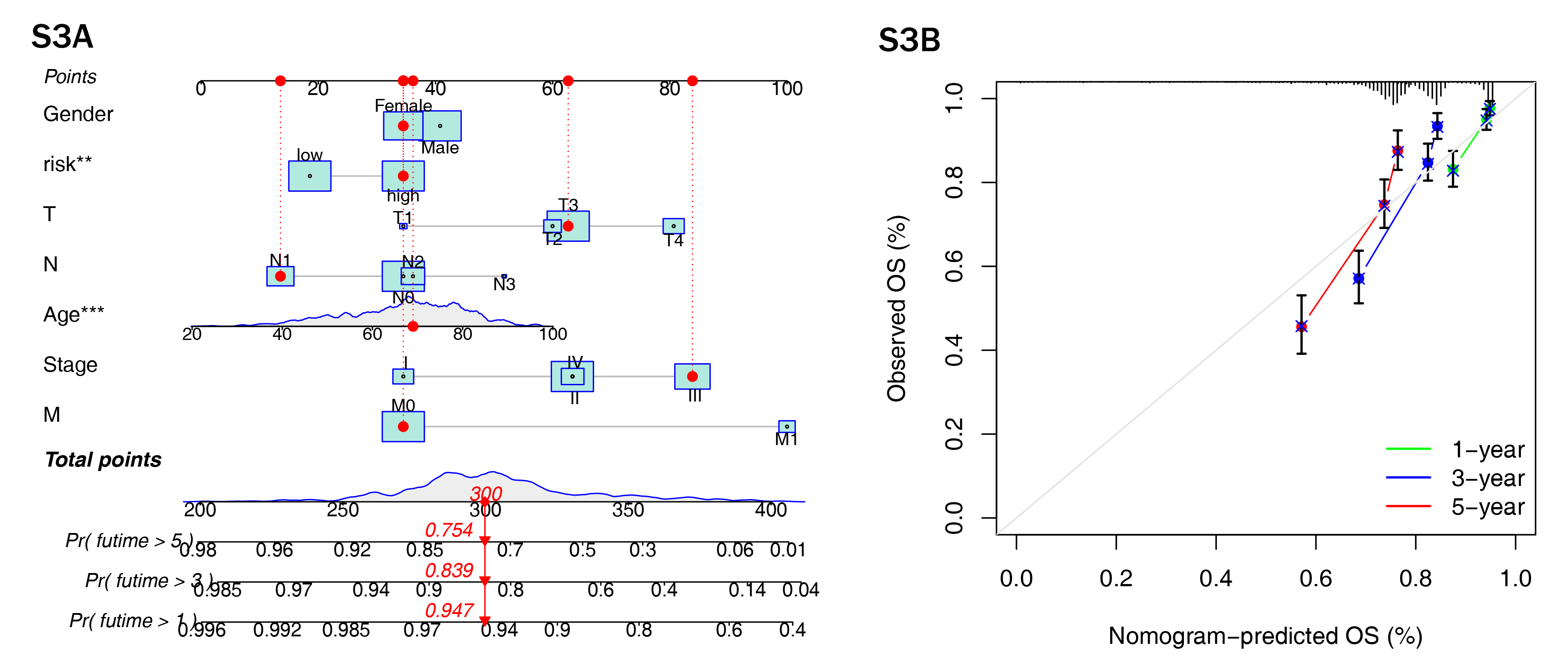

Supplement: Supplementary file 1 [file Image3.TIF]

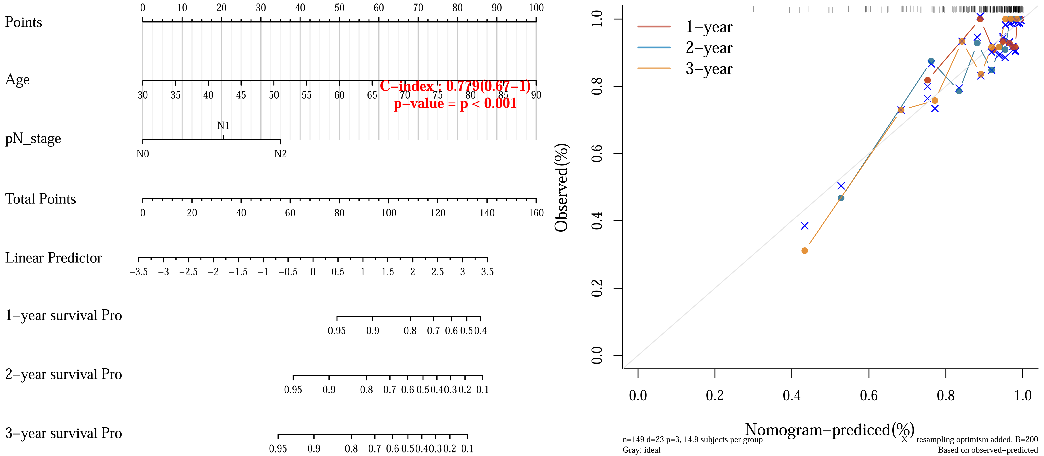

Supplement: Supplementary file 2 [file Image4.TIF]

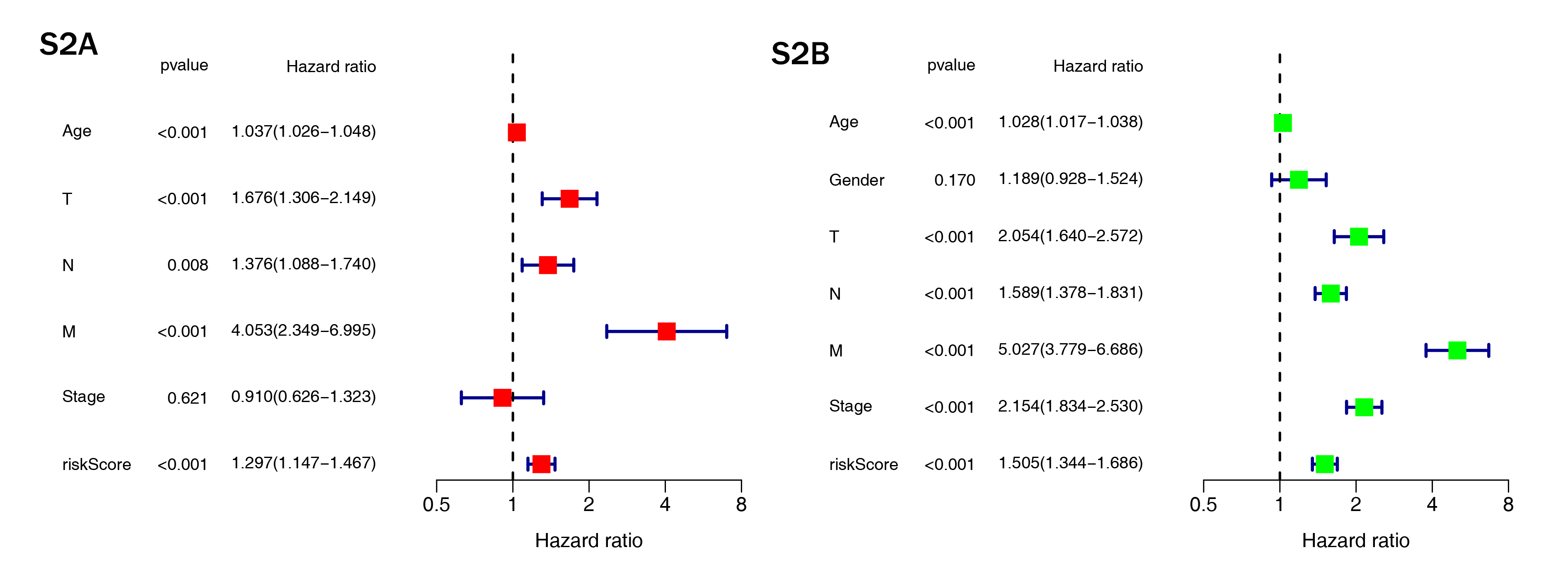

Supplement: Supplementary file 3 [file Image2.TIF]

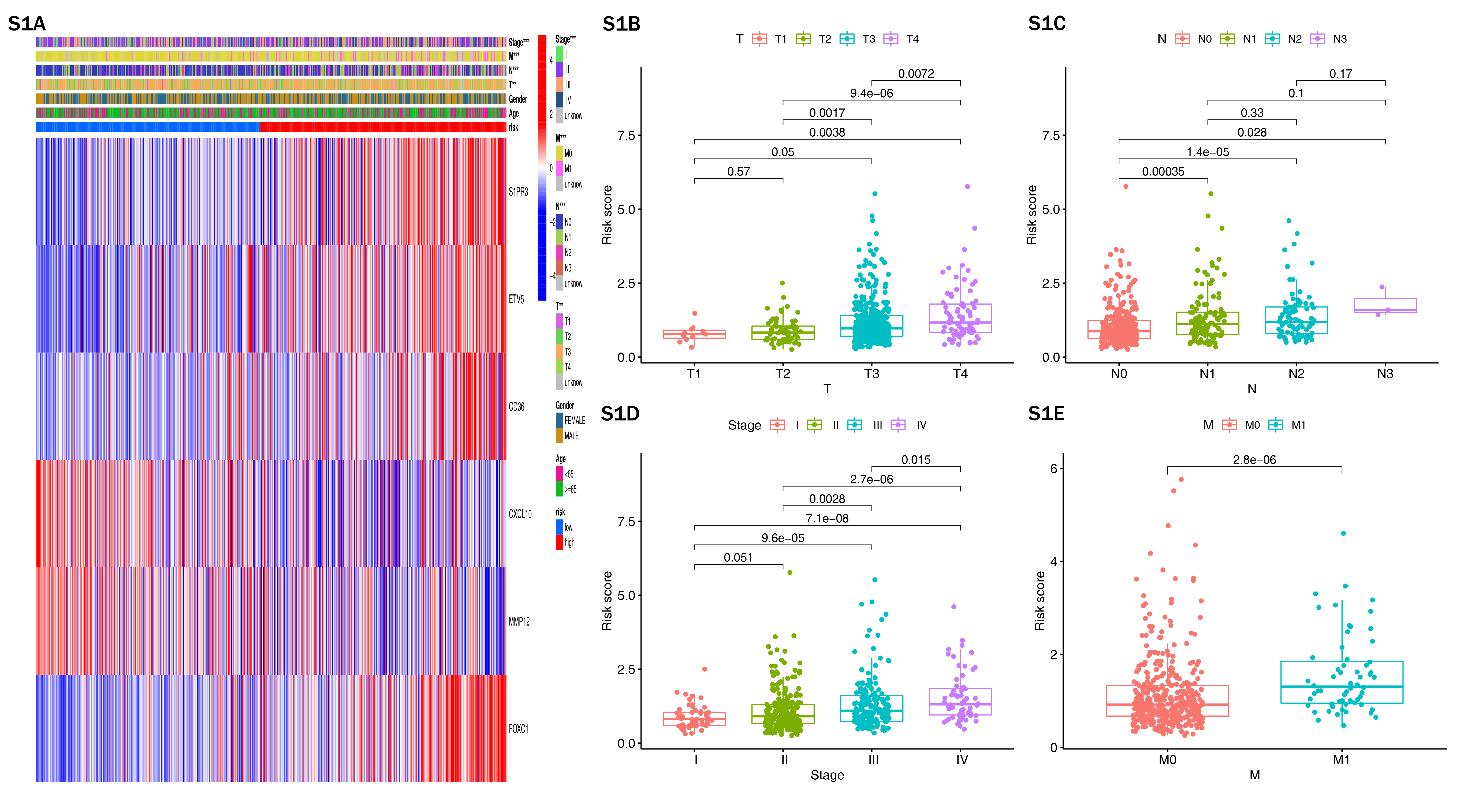

Supplement: Supplementary file 4 [file Image1.TIF]
